# Multiple Roles of Transforming Growth Factor Beta in Amyotrophic Lateral Sclerosis

**DOI:** 10.3390/ijms21124291

**Published:** 2020-06-16

**Authors:** Mariarita Galbiati, Valeria Crippa, Paola Rusmini, Riccardo Cristofani, Elio Messi, Margherita Piccolella, Barbara Tedesco, Veronica Ferrari, Elena Casarotto, Marta Chierichetti, Angelo Poletti

**Affiliations:** Dipartimento di Scienze Farmacologiche e Biomolecolari, Centro di Eccellenza sulle Malattie Neurodegenerative, Università degli Studi di Milano, 20133 Milano, Italy; valeria.crippa@unimi.it (V.C.); paola.rusmini@unimi.it (P.R.); riccardo.cristofani@unimi.it (R.C.); elio.messi@unimi.it (E.M.); margherita.piccolella@unimi.it (M.P.); barbara.tedesco@unimi.it (B.T.); veronica.ferrari@unimi.it (V.F.); elena.casarotto@unimi.it (E.C.); marta.chierichetti@unimi.it (M.C.)

**Keywords:** ALS, TGFB, motor neuron, glial cells, skeletal muscle

## Abstract

Transforming growth factor beta (TGFB) is a pleiotropic cytokine known to be dysregulated in many neurodegenerative disorders and particularly in amyotrophic lateral sclerosis (ALS). This motor neuronal disease is non-cell autonomous, as it affects not only motor neurons but also the surrounding glial cells, and the target skeletal muscle fibers. Here, we analyze the multiple roles of TGFB in these cell types, and how TGFB signaling is altered in ALS tissues. Data reported support a crucial involvement of TGFB in the etiology and progression of ALS, leading us to hypothesize that an imbalance of TGFB signaling, diminished at the pre-symptomatic stage and then increased with time, could be linked to ALS progression. A reduced stimulation of the TGFB pathway at the beginning of disease blocks its neuroprotective effects and promotes glutamate excitotoxicity. At later disease stages, the persistent activation of the TGFB pathway promotes an excessive microglial activation and strengthens muscular dysfunction. The therapeutic potential of TGFB is discussed, in order to foster new approaches to treat ALS.

## 1. Introduction

Amyotrophic lateral sclerosis (ALS) is a neurodegenerative disorder affecting the motor system. Nevertheless, it implicates different cell types: motor neurons are the main affected cells, but glial and skeletal muscle cells are strongly implicated and able to deeply modulate the disease onset and course. The neuromuscular system is a complex network; one of the most important factors influencing its development and maintenance is the transforming growth factor beta (TGFB), a pleiotropic molecule also known to be dysregulated in ALS patients. The aim of this review is to summarize the current knowledge of the different roles of TGFB in ALS. We will briefly describe why ALS is also considered a non-cell-autonomous disease, and the general features of the TGFB family; then, we will analyze the roles of TGFB on the multiple cell types involved in ALS, and we will discuss the TGFB pathway as a potential pharmacological target. We will only briefly mention the multiple effects of TGFB on the immune system in ALS, as it is a field that deserves a separate discussion, due to the various and multifaceted roles exerted by TGFB on this system.

## 2. Amyotrophic Lateral Sclerosis as a Non-Cell-Autonomous Disease

ALS is a disease affecting upper and lower motor neurons, with an incidence of 1–2/100,000 per year, and mean survival of 3–5 years after diagnosis [1]. ALS can occur in two different forms, sporadic (sALS, 90–95% of cases) and familial (fALS, 5–10% of cases), and it can also appear as a pure motor phenotype or in association with fronto-temporal dementia (ALS-FTD). ALS is characterized by a progressive loss of motor neurons, but the precise pathological mechanisms involved are not clear as their complex interplay with neighboring and target cells. Mutations in many different genes have been associated with fALS, starting from the superoxide dismutase 1 (*SOD1*) gene (coding for an antioxidant enzyme), to the more recently described mutations in TAR DNA-binding protein 43 (*TDP-43*), ALS-linked fused in sarcoma/translocated in liposarcoma (*FUS/TLS*), and other genes (see, for review [2]). Recently, 50% of fALS cases have been associated with an alteration of the chromosome 9 open reading frame 72 (*C9orf72*), resulting in the expansion of the hexanucleotide (G_4_C_2_) repeat located in its 5’-untranslated region [3,4,5].

ALS is primarily caused by the death of upper and lower motor neurons. Nevertheless, in the last 15 years, besides the main classical “neuron-centric” view of ALS, a number of research studies evidenced that ALS could also be a non-cell-autonomous disease [6,7]. Data have been mostly obtained using ALS mouse models, but they may also be linked to sALS cases [8]. Glial and skeletal muscle cells demonstrated their ability to trigger, or modulate, ALS. The analysis of chimeric mice indicated that the restricted expression of human mutant SOD1 (mutSOD1) in motor neuron is not sufficient to induce a cell-autonomous degeneration of motor neurons [9]. Moreover, utilizing floxed mutSOD1 gene, it has been demonstrated that the damaging process starts in motor neurons and determines the disease onset, with little influence on its progression [6]. Conversely, mutSOD1 activates glial cells exacerbating the disease progression, while motor neuronal mutSOD1 has little influence on the progression of ALS [6].

Among the non-neuronal neighbors of ALS motor neurons, glial cells are the most investigated, so far. Astrocytes, microglia, oligodendrocytes, and Schwann cells are all able to modulate ALS pathology, and gliosis is a hallmark of ALS from an immuno-histological point of view (see, for review [10,11]).

Activated and proliferating astrocytes become neurotoxic, and no longer provide the metabolic support to motor neurons, but secrete cytokines or other toxic factors (among which is the TGFB) that are critical for determining the rate of disease progression [12,13]. Furthermore, activated astrocytes reduce the expression of the excitatory amino acid transporter-2, that is mandatory for glutamate re-uptake from the synaptic cleft into astrocyte, leading to excitotoxicity in motor neurons [14]. 

Microglia have long been known to be activated in ALS affected tissues, probably through the innate immune system. The extent of its activation correlates with the severity of the upper motor neuron involvement [15]. The non-cell-autonomous mechanisms of toxicity of microglia include the secretion of pro-inflammatory cytokines [16]. The reduction of mutSOD1 toxicity within microglia slows the progression of the disease, suggesting that microglia might contribute to the neurodegenerative processes of ALS [17]. Other studies indicated that a decreased number of microglial cells is present at the pre-symptomatic stage in mouse models, while two distinct microglia populations can be identified after symptom manifestation [18]. Whether microglial cells are beneficial or detrimental to motor neurons is already an open question. Probably, they exert a surveillance role on motor neurons and restore the correct environment after an injury but, when constitutively activated in the presence of a chronic stress (such as that causing ALS), they may become toxic.

Other glial cell types are important for motor neuron functionality. Oligodendrocytes provide metabolic support to central nervous system (CNS) neurons and support their survival, while Schwann cells are strictly related to motor neuron axons and support axonal development and regeneration in the peripheral nervous system (PNS). Recent studies implicate oligodendrocytes in ALS pathogenesis, through both myelin sheath disruption and the reduction of the monocarboxylate transporter 1 [19]. Furthermore, it has been shown that oligodendrocytes isolated from human ALS patients are able to induce motor neuron death in a co-culture system [20]. Regarding Schwann cells, few studies addressed their role in ALS, suggesting that axonal degeneration may occur earlier than myelin degeneration. 

In addition to neighboring cells, motor neurons can also be influenced by their target, the skeletal muscle cells. It has been shown, at least in fALS, a direct muscular toxicity and/or a functional impairment that has denervation and motor neuron death as a consequence [21,22,23]. Muscle dysfunction and neuromuscular junction (NMJ) degeneration occur long before disease onset [24]. A contribution to the initiation and progression of muscle atrophy is given by altered ALS satellite cell properties [25,26]. Furthermore, muscle gene expression is changed since the early stages of the disease [27,28,29]. Regarding the presence of muscle dysfunction in relation to the expression of other mutant genes linked to ALS, it must be recalled that TDP-43 immuno-reactivity is detectable only in muscle fiber nuclei without any sarcoplasmic TDP-43 aggregation [30,31]; very recently, it has been demonstrated that skeletal muscle contributes to the ALS phenotype also in *C9orf72* related cases [32]. These authors demonstrated that the presence and the amount of dipeptide repeats in patient’s muscles are significantly related to a more severe muscular atrophy. In addition, our previous works have indicated that the protein quality control system is a dysfunctional cellular process in ALS muscle cells, but these cells seem to be more protected than motor neurons against the presence of accumulating misfolded proteins [33,34,35]. Proteasome activity is impaired by mutSOD1 only in motor neurons and not in muscle cells [35]. Nevertheless, we proved that motor neurons are characterized by a higher autophagic potential with respect to muscle cells. These results could help to clarify why muscle cells seem more protected than motor neurons from misfolded SOD1. Parallel results have indicated that muscle cells mainly depend on the proteasome system to quickly remove misfolded TDP-43 [33]. As a whole, data indicate that autophagy modulation could be a potential therapeutic approach to counteract muscle atrophy in ALS and to promote aggregate removal in motor neurons.

## 3. Transforming Growth Factor Beta

TGFB is a family of cytokines with widespread and diverse effects. During development and in adulthood, TGFB family member signals can reach practically all the cells modulating their activities [36]. The TGFB superfamily comprises 32 members grouped into different families, including TGFB, activin, growth and differentiation factor (GDF), and bone morphogenetic protein (BMP) families (Table 1) [37]. Among all these ligands, TGFB1 and myostatin are considered the most implicated in skeletal muscle development and function, with shared or contraposed features.

**Figure 1:**
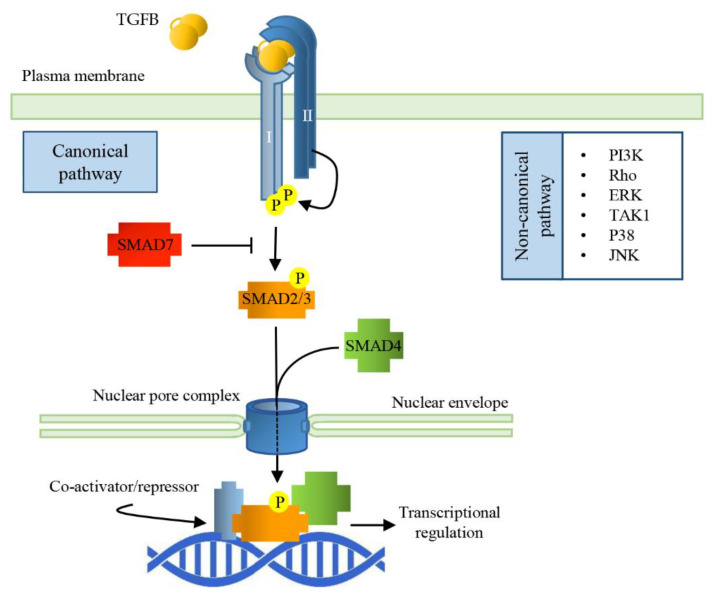
Signal Transduction pathways of TGFBs. TGFB dimerization triggers the assembly of a heterodimeric complex between Type I and Type II receptors (TGFBRI and TGFBRII). This permits TGFBRII to trans-phosphorylate TGFBRI that, in turn, activates the receptor-regulated SMADs (SMAD2/3) by phosphorylation. Activated R-SMAD forms a complex with the common SMAD (SMAD4) and together translocate into the nucleus through nucleoporins; the complex interacts with specific SMAD binding elements (SBEs), regulating gene transcriptional responses.

The bioactive TGFB ligands are disulfide-linked dimers cleaved from the C-terminal portion of a precursor. Usually, the ligands are homodimeric, but also heterodimers exist. All the ligands of the TGFB family bind to two pairs of receptors that are transmembrane serine/threonine protein kinases. The binding of the cytokine to the type II receptor (TGFBRII) leads to its activation, and to the phosphorylation of the type I receptor (TGFBRI), allowing it to phosphorylate small mother against decapentaplegic (SMAD) transcription factors which shuttle between the cytosol and the nucleus [36]. In the nucleus, SMADs bind to specific responsive elements (SMAD binding elements, SBEs) throughout the genome, activating or repressing a variety of different responsive genes (Figure 1). 

There are eight SMAD proteins in mammals: five are receptor regulated (R-SMAD, SMAD1, 2, 3, 5, 8), two are inhibitory (SMAD6 and 7), and one (SMAD4) is a protein common to all the pathways of TGFB family members. In the pathway activated by TGFB ligands, the TGFBRI phosphorylates SMAD2 and SMAD3. Receptor-mediated phosphorylation facilitates oligomerization between R-SMADs and SMAD4. The formation of this complex is mandatory to bring the signal from the cytosol to the nucleus, but, so far, SMAD4 specific function is unknown since certain TGFB functions do not require SMAD4. SMADs nuclear translocation does not depend on nuclear transport factors or importins; they can directly interact with nucleoporins [38]. The SMAD dependent signaling pathway of TGFB works ubiquitously in all cell types; however, in a context dependent manner, TGFB can activate SMAD-independent signaling cascades, such as PI3K, MAPK, or small GTPases [39]. Inhibitory SMADs bind an already activated TGFBRI, leading to the inhibition of the R-SMAD phosphorylation and their subsequent translocation into the nucleus and adding a further level of regulation of the TGFB/SMAD signaling cascade.

Once in the nucleus, SMAD complexes target specific promoters to regulate gene expression and microRNA processing. R-SMADs can directly bind DNA through SBEs giving gene specificity to the complex, while SMAD4 promotes or inhibits transcriptional activity, recruiting different, tissue-specific co-regulators [40]. Phosphorylated SMAD (pSMAD) signaling is terminated through phosphatase (PPM1A/PP2Cα)-mediated dephosphorylation and SMAD export from the nucleus to the cytosol. Alternatively, nuclear pSMADs are targeted for ubiquitination and subsequent cytosolic proteasomal degradation [41]. 

TGFB signaling can also be “non-canonical”; in this case, its effects are transduced by Smad-independent pathways, which include ERK MAPKs, a TGFB-activated kinase 1 (TAK1 activating JNK, p38K, and NF-κB), and PI3K-AKT [42]. The differential activation of non-SMAD pathways is context dependent; for example, in myotubes, the atrophic effect of TGFB seems to be linked to ERK1/2 and JNK1/2, in addition to SMADs [43].

TGFB regulates a plethora of cellular functions, in different contexts, ranging from embryonic development to tumor progression, from immune regulation to tissue fibrosis, and neurotrophic response modulation. Dysregulation of the TGFB pathway has been reported also as a common feature in neurodegenerative disorders, and among them, in pathologies affecting motor neurons, and particularly in ALS. The following paragraphs will summarize alterations in the TGFB pathway reported in many different ALS models, both in vivo and in vitro, in addition to those detected in patients. 

## 4. TGFB Plasma Levels in ALS Patients

Similarly to Parkinson’s and Alzheimer’s patients [44,45], in which an increased concentration of TGFB1 was found in the cerebrospinal fluid (CSF) or serum respectively, TGFB1 plasma concentration in ALS patients is significantly higher than in the healthy controls, and it positively correlates with the disease [46]. Indeed, TGFB1 is increased in the serum of ALS patients at an advanced stage of disease; likewise, TGFB1 is also augmented in the CSF of ALS patients with long disease duration [47]. A recent study has confirmed the existence of a negative correlation between TGFB1 and TGFB3 levels and ALS severity; this study postulated that high levels of TGFB in the serum might represent a compensatory mechanism to counteract the pronounced systemic immune response typical of the late stage of the disease, by inducing T cells to differentiate into non regulatory phenotypes [48]. Even if the increase in plasma TGFB1 levels has been confirmed also in mutSOD1 transgenic mice [49], whether TGFB1 plasma levels are biomarkers of ALS or not is still an open question; in fact, other studies fail to detect the changes of TGFB1 levels in patient CSF compared to healthy controls [50], or between fast and slow progressing ALS patients analyzed both at early and late stage of disease [49].

In the attempt to find specific ALS-susceptibility genes, a single-nucleotide polymorphism in the *ZNF512B* gene has been identified; the *ZNF512B* gene codes for a transcription factor with a reduced ability to promote TGFB signaling [51]. A retrospective analysis of this gene in ALS patients indicated a significantly lower probability of survival in patients, carrying the risk allele independently from other factors know to be involved in ALS [52]. For this reason, *ZNF512B* might be a new prognostic factor in ALS.

## 5. TGFB and ALS-Nervous System

In the adult rodent nervous system, TGFB1 immunoreactivity is constitutively present only in meninges and the choroid plexus in the brain [53,54]. However, TGFB1 mRNA is more widely expressed, with intense labeling in cortical layers 2, 3, and 5, hippocampus, retinal ganglionic cells, some hypothalamic areas, and the ventral horn of the spinal cord [54,55]. TGFB2 and 3 are widespread and distributed [53,54]. The immunoreactivity of TGFB is also present in astroglial cells. The expression of TGFBRI and TGFBRII has been demonstrated in neurons, astrocytes, oligodendrocytes, microglia, and brain endothelial cells [56,57].

**Figure 2:**
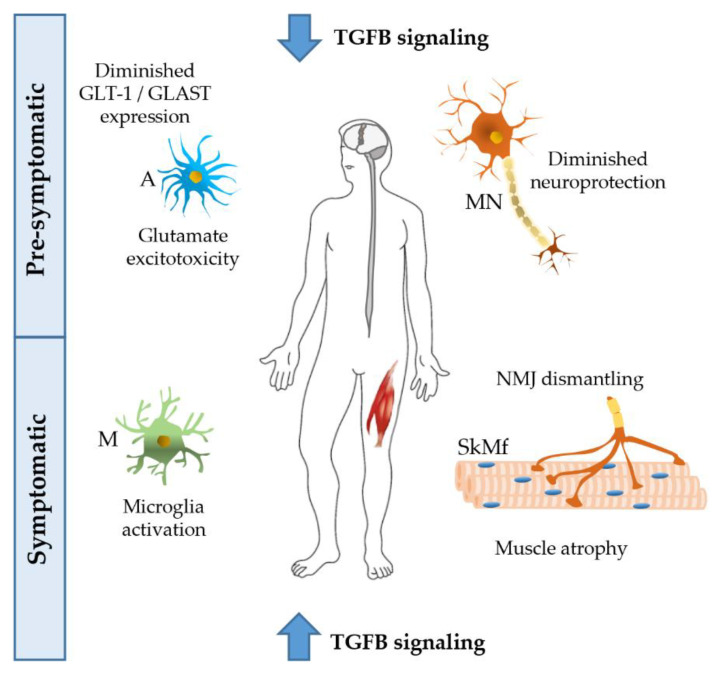
Possible effects of TGFB on different tissues of amyotrophic lateral sclerosis (ALS) patients. At the pre-symptomatic stage, the decreased activation of the TGFB pathway reduces its neuroprotective activity, and, at the same time, increases excitotoxicity induced by glutamate, with a lesser uptake by astrocytes. At the symptomatic stage, TGFB levels are largely increased, giving rise to microglia activation, and neuromuscular junction (NMJ) dismantling, thus leading to atrophy of skeletal muscle fibers. ALS disease progression could be promoted by a chronically altered TGFB pathway. A, astrocyte; M, microglia; MN, motor neuron; SkMf, skeletal muscle fibers.

TGFBs have multiple functions in the CNS. They enhance synapse formation and synaptic transmission [58,59], regulate synaptic plasticity and memory [57], increase the number and length of neurites [56], control neuronal migration [60], and cerebral cortex angiogenesis [61]. CNS-TGFB1-deficient mice have a reduced brain weight and a loss of neurons in the CA1 hippocampal region. These mice show a reduction of dendritic spine density, impaired long-term potentiation, and facilitated long-term depression in the hippocampus, in addition to the loss of the astrocyte glutamate transporters GLT-1 (EAAT2) and GLAST (EAAT1), and decreased glutamate uptake, resulting in a higher sensibility to glutamate excitotoxicity, that is one of the possible pathogenic mechanism in ALS (Figure 2) [62].

Motor nerve terminals show an intense TGFBRI and II immunoreactivity [63]; thus, they are expected to be responsive to TGFB. Furthermore, the Schwann cell side of the synapse and axons express TGFBRII, suggesting that motor neurons are exposed to TGFB along their full length. Indeed, motor neurons are surrounded by different cell types, all capable of producing and releasing TGFB: blood cells, Schwann cells, muscle fibers. During development, TGFB promotes motor neurons survival and saves them from naturally occurring cell death, due to competition for a limited amount of survival factors provided by all the cells that they interact with [64]. In primary motor neuron cultures, TGFB1 protects cells from damage caused by cytotoxic hypoxia or excitatory amino acids inducing an increase in cell viability, neuronal ATP levels, and protein content [65]. In vivo, its administration attenuates axotomy-induced motor neuron death, even if its rescue effect is not permanent [66]. TGFB2 is a motor neuron survival factor concentrated in the post-synaptic domain of mature rodent and human muscle fibers [67]. Nevertheless, in specific conditions, motor neurons are able to produce TGFB2 in an autocrine manner, probably to counteract peripheral (dendritic, or at the nerve terminal) apoptotic signals [63]. 

Even if the comparative analysis of fALS and sALS tissues indicates the existence of common and distinct biological mechanisms driving the different forms of the pathology, altered levels of the TGFB1 pathway have been reported in motor neurons of most ALS models and patients. Recently, an analysis of DEGs and DEPs in induced pluripotent stem cells (iPSC)-derived motor neurons from patients with mutations in *C9orf72* indicated TGFB and SMAD2/3 targets among the pathway most involved with a high correlation between significantly altered mRNA and proteins [68]. Similar results have been obtained in postmortem spinal motor neurons from sALS patients. The mutated TDP-43 protein aggregates form intracytosolic inclusion bodies that sequester pSMAD2/3 and SMURF2, an E3 ubiquitin ligase promoting the ubiquitin-dependent degradation of SMAD proteins, suggesting an impaired TGFB signal in motor neurons of sALS patients [69,70]. Interestingly, TDP-43 inclusions of brain extra-motor neurons do not co-localize with pSMAD2/3, and its nuclear staining is preserved, indicating regional differences in the composition of the inclusions and in the impairment of TGFB signaling [70].

An excessive activation of the TGFB pathway has also been reported in ALS4 patients characterized by mutation in senataxin (*SETX*) gene. These patients present fewer R-loops (three-stranded nucleic acid structures) and their differentially-expressed genes are highly enriched for activation of the TGFB pathway [71]. Principally, it is decreased in the expression of BAMBI, a TGFB pseudo receptor, which lacks the intracellular kinase domain and acts as a negative regulator, leading to an increased TGFB signaling. Indeed, in the same ALS4 patients, levels of pSMAD2/3 are increased in the anterior horn of the spinal cord [71].

TGFB/SMAD targets are abnormally regulated in iPSC-derived motor neurons from patients with mutSOD1, even if, in this case, they belong to downregulated gene sets [68]. Neuronal injuries, such as oxidative stress, rapidly up-regulate *TGFB1* mRNA, inducing the expression of multiple genes involved in neuronal protection, and counteracting neuronal damage [72]. Reduced *Tgfb1* mRNA levels in the spinal cord of pre-symptomatic mutSOD1 mice could indicate a lack of the TGFB neuroprotective effect in the early stages of the disease [29]. With disease progression, *Tgfb1* gene expression increases in the spinal cord, probably for the development of reactive astrogliosis [29,73]. In fact, it must be highlighted that all types of glial cells are able to produce and/or respond to TGFB. The role of glia-derived TGFB1 in the spinal cord of ALS patients and mice has been studied by Endo and colleagues [12]. They observed an upregulation of TGFB1 in the lumbar spinal cord of ALS mice, mainly in astrocytes. Furthermore, *TGFB1* mRNA levels negatively correlated with the survival of ALS mice. Taking advantage of the ALS mice overexpressing TGFB1 in astrocytes and of the ALS mice with the astrocyte-specific deletion of TGFB1, they determined that astrocyte-derived TGFB1 accelerates disease progression in ALS mice, preventing neuroprotective responses mediated by the microglia and T cells [12].

In the spinal cord of ALS mice, the TGFB signaling pathway is also altered. *Tgfbr2* mRNA levels are increased [29] in agreement with other authors, indicating a higher TGFBR2 immunoreactivity [73], and higher levels of *Tgfbr2* in human and mouse spinal cord ALS samples [74,75]. 

In mutSOD1 mice, levels of pSMAD2 in the nuclei of lumbar motor neurons are significantly decreased already at the pre-symptomatic stage, while they are preserved at the cytosolic level as the expression of TGFBR1 and 2 [12,69,76]. This led to the hypothesis of an aberrant nucleo/cytosolic transport with an accumulation of cytosolic pSMAD2/3 immunoreactivity [69,76]. Moreover, the presence of pSMAD2 in glial nuclei is preserved. This hypothesis is also supported by the fact that physiological pSMAD2 levels in the nucleus are not recovered by overexpressing TGFB1 [12], and that the expression of *Smad2* and *Smad4* is decreased [29]. Otherwise, an up-regulation of SMAD4 in the spinal motor neurons of autopsied sALS cases has also been reported [77].

## 6. TGFB Pathway in ALS Skeletal Muscle

In skeletal muscle, the expression of TGFB is related to normal processes such as growth, differentiation, regeneration, and stress response. However, continuously elevated levels of TGFB are linked to impaired regeneration and atrophy. TGFB blocks myogenic responses and stimulates fibrosis [78]. It inhibits the activation of MyoD and myogenin (two transcription factors regulating muscle cell differentiation) through the signaling of SMAD3 or by inactivating cyclin-dependent kinases [79,80]. Satellite cell activation is also prevented in the presence of TGFB, and muscle overexpression of TGFB leads to muscle weakness and atrophy [81,82]. Furthermore, TGFB has a dual role in the inflammatory process taking place after muscle injury: it acts first as a stimulating factor, and then as a suppressor for muscle inflammatory response. Injured muscle fibers secrete TGFB, and in different disorders, its muscle levels are elevated [83]. Spinal and bulbar muscular atrophy (SBMA), a polyglutamine disease dependent on the expansion of the CAG repeat within the androgen receptor (AR) affects motor neurons and skeletal muscles and it is also associated with the disruption of TGFB signaling [84,85]. Mdx mice (a model of Duchenne muscular dystrophy) are characterized by myofiber degeneration and augmented TGFB1 signaling [86]. Furthermore, in this murine model, the neutralization of TGFB1 signaling may enhance the differentiation and fusion of the precursor satellite cells, suggesting a direct role for this cytokine in skeletal muscle regeneration [87]. 

ALS muscle tissue is also characterized by alterations of the TGFB pathway. We reported increased levels of the *Tgfb1* mRNA in the muscle of mice expressing mutSOD1 [28]. These changes are also gender-related, since male mice present an increased TGFB expression in muscle already at the pre-symptomatic stage, both at the mRNA and protein level, while in female animals, TGFB increases only at the symptomatic stage [29]. *Tgfb* mRNA levels are further increased with the administration of an anabolic/androgenic steroid (AAS), indicating that, at least at the muscular level, AAS might exert a detrimental role in ALS, since it might exacerbate some of the alterations induced by mutSOD1 [28,88]. Moreover, data obtained with the C2C12 muscle cell model indicate that an increase of the AR (that was obtained in vivo by stabilizing the receptor through a chronic AAS treatment) may also modify the effect of the wild type human SOD1, leading to an augmented TGFB1 expression [28]. These data might help to explain gender differences in the risk to develop ALS [2]. Evidence in human confirmed the involvement of TGFB1 since we reported an increased *TGFB1* expression in muscle of female and male sporadic ALS patients with a significant gender effect [29], and other authors also reported the increase of *TGFB1*, *2*, and *3* in ALS patient muscles [89,90]. It must also be highlighted that *TGFB1* and *TGFB3* mRNA show a negative correlation with muscle strength in ALS patients [90]. In the same manner, the increase of TGFB1 correlates with disease progression in mutSOD1 mice [28] and *Smad1*, *5*, and *8* expression negatively correlates with mouse rotarod scores [91]. As a result, TGFB1 has been proposed as a possible biomarker of ALS progression [29,90,92].

The analysis of muscle lysates from ALS patients indicated a strong increase of TGFB1 protein [90]. Even if, in these samples, TGFB1 immunoreactivity has been detected in macrophages and lymphocytes surrounding the fibers and suggesting inflammatory infiltrated cells as the TGFB1 source, we reported that the expression of mutSOD1 in C2C12 cells stimulates *Tgfb1* expression [28], and C2C12 cells are able to respond to TGFB, increasing SMADs production and phosphorylation [90].

Regarding the intracellular pathways mediating TGFB functions in muscle cells, different works have evidenced changes in the levels of SMAD proteins. We reported an increased expression of SMAD2 and SMAD3 in symptomatic ALS mice, in agreement with Si and collaborators [29,90]. Evidence in ALS patients is more contradictory, reporting increased [89], diminished [29], or unchanged [90] *SMADs* muscle expression. These discrepancies might be due to the heterogeneity of ALS patients (sporadic vs. familiar), to the site of the biopsies (deltoid, tibialis anterior, vastus lateralis), duration of the pathology, site of onset, etc. R-SMADs enter into the nucleus through SMAD4, and in line with the work of Saris et al., we found a decreased *Smad4* expression in muscle, suggesting a further site of dysregulation of TGFB intracellular signaling [93]. The involvement of other SMAD proteins (SMAD 1, 5, and 8, usually more involved in the BMP signaling pathway), has been reported [91]. BMP is one of the strongest hypertrophic signals in muscle; for this reason, the increase of these SMADs could represent a way to counteract denervation-induced atrophy. Whether SMAD changes are related to the progressive loss of motor neuron innervation or to a muscle pathological modification contributing to its atrophy and to disease progression has yet to be established. However, we reported that mutSOD1 toxicity can be exerted independently of its tendency to aggregate. At motor neuron level, mutSOD1 forms proteinaceous inclusions that alter SOD1 protein bioavailability and turnover [94] and reduce the protective effect exerted by wild type SOD1 against free radical reactive oxygen species [95]. On the contrary, in the gastrocnemius muscle of ALS mice, an increase of mutSOD1 levels was detected only at the terminal stage, and no high molecular weight SDS-resistant species of mutSOD1 were identified, either in ALS mice or in C2C12 cells expressing mutSOD1 [28,35].

The hypothesis of an impairment of TGFB signaling in motor neurons at the step of pSMAD 2/3 translocation into the nucleus is also supported by data obtained in the muscle since, usually, TGFB1 inhibits *MyoD* transcription through SMAD3, while in mutSOD1 mouse muscle, and in mutSOD1 transfected C2C12 cells, we detected high levels of *MyoD* mRNA [28].

It has also been proposed that excessive oxidative processes may be a mechanism of activation of latent TGFB pool in ALS, as in other neurodegenerative diseases, leading to an increased TGFB1 release from the complex [96].

TGFB1 is a potent inducer of fibrotic tissue formation, promoting the transformation of myoblasts into fibrotic tissue after an injury, inhibiting satellite cell activation, and impairing myocyte differentiation [86]. In the skeletal muscle of mutSOD1 expressing mice fibrosis is enhanced and correlates with TGFB levels, therefore, we can hypothesize that the beneficial effects of reducing TGFB levels could also be associated with a reduction of fibrosis [97].

## 7. TGFB and NMJ in ALS

Since the first histological studies, recurrent denervation and reinnervation have been observed in the NMJs of ALS patients [98]. Because of that, it has been proposed to consider ALS also as a distal axonopathy, with pathological changes occurring at the NMJs prior to motor neuron degeneration and muscle fiber atrophy (see, for review [99]).

TGFB pathway regulates the formation and stability of the NMJs. All the members of this tripartite synapse are able to produce and/or respond to TGFB. Muscle fibers express TGFB1, motor neuron terminals express TGFB receptors, and the synapse associated Schwann cells, also called terminal Schwann cells (TSCs, which are active components of the NMJ [100]), promote synaptogenesis at the NMJ via TGFB1 [101]. In this site, TGFB1 is capable of doubling the size of acetylcholine receptor clusters increasing the percentage of nerve–muscle contacts. It has also been demonstrated that this synaptogenic effect of TGFB1 might be ascribed to its ability to induce neuronal agrin expression [101]. Agrin is a proteoglycan important for the maintenance of the architecture of the postsynaptic membrane, known to be down-regulated in the muscle of ALS mice expressing mutSOD1 [22].

Recent studies indicate that alterations of the TSCs are present well before motor neuron terminal degeneration and the beginning of denervation in mutSOD1 expressing mice [102]. An anomaly appears as the absence of TSCs at many NMJs of the fast medial gastrocnemius, and with TSC cell body displacement at other NMJs of gastrocnemius or soleus muscle. This evidence could support the different extent of denervation between fast and slow muscles [102].

Among the factors secreted by muscle fibers and concentrated at the NMJ to promote synapse well-being, there is the fibroblast growth factor binding protein 1 (FGFBP1) [103]. FGFBP1 is a secreted factor that might potentiate the bioactivity of different members of the FGF family during reinnervation, by releasing sequestered FGF from the extracellular matrix [103]. The expression of this binding protein is known to be decreased in mutSOD1 mouse skeletal muscles before NMJ degeneration, and the deletion of FGFBP1 from mutSOD1 mice accelerates NMJ degeneration and disease progression [104]. The same authors reported also that TGFB1 is highly concentrated at NMJs of pre-symptomatic mutSOD1 mice, and represses FGFBP1 expression, indicating TGFB1 pathway as a potential target for preventing NMJ dismantling in ALS mice [104].

Moreover, other genetic models of ALS present NMJ modifications. For example, the loss of function of VAPB in Drosophila reduces the number of boutons that are also larger than in wild type flies, and with a highly disorganized microtubule network [105]. Again, a Drosophila ALS model expressing a mutant form of an ortholog of VAPB (resembling the loss of function phenotype), shows reduced pMAD (an ortholog of SMAD) at the NMJ and in CNS [106].

## 8. TGFB: A Target for ALS Treatment 

The therapeutic potential of TGFB has been investigated, in order to develop new therapies for ALS. 

In a study performed with mutSOD1 expressing mice, SB-431542, a selective inhibitor of TGFBRI kinase activity, has been proven to extend animal survival, even if administered after disease onset; this led the authors to hypothesize that TGFB1 produced by astrocytes inhibits the neuroprotective response given by microglia and inflammatory cells and could be considered a negative prognostic factor [12]. 

Moreover, the intraperitoneal injection of TGFB2 is able to acutely improve the motor performance of ALS mice. It reverses initial muscle weakness, permitting a better performance at rotarod test, probably through a marked trophic action on motor neurons, as can be inferred by motor neuron nuclei and axonal enlargement. Unfortunately, this advantage is transient, leading to an even more rapid progression of the disease [107]. 

Antibodies neutralizing other members of the TGFB family have been tested in ALS models. For example, the prevention of myostatin binding to its receptor delayed the onset and the progression of the disease in ALS mice, even if without extending their survival [108,109].

## 9. Conclusions

The pleiotropic effects of TGFB in ALS have been analyzed and are summarized in Figure 2.

The imbalance of TGFB signaling has been linked to ALS progression. On one hand, at the level of the CNS, there is a lack of the neuroprotective effects of TGFB at the first stages of the disease; later, the strong increase of TGFB levels due to microglial stimulation shifts the CNS milieu toward a proinflammatory and neurotoxic environment. On the other hand, at the level of the skeletal muscle, the chronically increased TGFB signaling facilitates the development of atrophy and fibrosis in skeletal muscle fiber, and the process of NMJ dismantling. Furthermore, the higher pre-symptomatic levels of TGFB in male vs. female muscle support the evidence that males are more vulnerable than females in ALS. Whether muscle effects are the cause or the result of the progressive motor neuron degeneration remains to be established.

Taken together, the data reviewed here support the hypothesis that the TGFB pathway may be considered critical for ALS etiology and progression. Thus, TGFB and its signaling pathway could represent a promising target for developing new therapies for ALS. 

## Figures and Tables

**Table 1:** Transforming growth factor beta (TGFB) family members, their receptors, and SMAD signaling proteins.

**TGFB Super Family**	**Family**	**Family Members**	**Type I Receptor**	**Type II Receptor**	**R-SMAD**	**I-SMAD**
	**TGFB**	**TGFB 1–5**	**TGFBR1**	**TGFBR2**	**SMAD2/3**	**SMAD7**
	**ACTIVINS/INHIBIN**	**ACTIVIN A, B**	**ACVR1B, ACVR1C**	**ACVR2, ACVR2B**	**SMAD2/3**	**SMAD7**
		**INHIBIN A, B**	**/**	**ACVR2**	**/**	**/**
		**LEFTTY A, B**	**/**	**/**	**/**	**/**
		**NODAL**	**/**	**ACVR2, ACVR2B**	**SMAD2/3**	**SMAD6/7**
	**BMP**	**BMP 2, 4**	**BMPR1A, BMPR1B**	**ACVR2, ACVR2B, BMPR2**	**SMAD1/5**	**SMAD6/7**
		**BMP 3**	**/**	**ACVR2B**	**/**	**SMAD6/7**
		**BMP 5–8**	**ACVR1A, BMPR1A, BMPR1B**	**ACVR2, ACVR2B, BMPR2**	**SMAD1/5**	**SMAD6/7**
		**BMP 9, 10**	**ALK1**	**ACVR2, BMPR2**	**SMAD1/5**	**SMAD6/7**
		**BMP 15**	**BMPR1B**	**BMPR2**	**SMAD1/5**	**SMAD6/7**
		**AMH**	**ACVR1A, BMPR1A**	**AMHR2**	**SMAD1/5**	**SMAD6/7**
	**GDF**	**GDF 1, 3**	**ACVR1B, ACVR1C**	**ACVR2, ACVR2B**	**SMAD2/3**	**SMAD7**
		**GDF 8 (MYOSTATIN)**	**ACVR1B, TGFBR1**	**ACVR2**	**SMAD2/3**	**SMAD7**
		**GDF 9**	**ACVR1B**	**BMPR2**	**SMAD2/3**	**SMAD7**
		**GDF 11**	**ACVR1B**	**ACVR2, ACVR2B**	**SMAD2/3**	**SMAD7**
		**GDF 5–7**	**BMPR1A, BMPR1B**	**ACVR2, ACVR2B, BMPR2**	**SMAD1/5**	**SMAD7**
		**GDF 15**	**GFRAL**	**/**	**/**	**/**

## Abbreviations

ALS	amyotrophic lateral sclerosis
SOD1	superoxide dismutase type 1
C9orf72	chromosome 9 open reading frame 72
TDP-43	TAR DNA-binding protein 43
CNS	central nervous system
PNS	peripheral nervous system
TGFB	transforming growth factor beta
TGFBRI	TGFB type I receptor
TGFBRII	TGFB type II receptor
SMAD	small mother against decapentaplegic
SBE	SMAD binding element
